# The Role of Nailfold Videocapillaroscopy in the Diagnosis and Monitoring of Interstitial Lung Disease Associated with Rheumatic Autoimmune Diseases

**DOI:** 10.3390/diagnostics15030362

**Published:** 2025-02-04

**Authors:** Daniela Anghel, Oana-Georgiana Prioteasă, Iulia-Nadine Nicolau, Săndica Bucurică, Daniela-Opriș Belinski, Gilda-Georgeta Popescu, Minerva Claudia Ghinescu, Anca Bobircă, Maria-Laura Groșeanu, Violeta-Claudia Bojincă

**Affiliations:** 1 Department of Internal Medicine 2, Central Military Emergency University Hospital ‘Dr. Carol Davila’, 010825 Bucharest, Romania; 2 Department of Medico-Surgical and Prophylactic Disciplines, ‘Titu Maiorescu’ University, 031593 Bucharest, Romania; 3 Department of Internal Medicine and Rheumatology, ‘Carol Davila’ University of Medicine and Pharmacy, 020021 Bucharest, Romania; 4 Department of Gastroenterology, Central Military Emergency University Hospital ‘Dr. Carol Davila’, 010825 Bucharest, Romania; 5 Department of Gastroenterology, ‘Carol Davila’ University of Medicine and Pharmacy, 020021 Bucharest, Romania; 6 Department of Rheumatology, ‘Sf. Maria’ Clinical Hospital, 011172 Bucharest, Romania; 7 Tuberculosis Control Subcomission, Romanian Ministry of Health, 030167 Bucharest, Romania; 8 Department of Internal Medicine and Rheumatology, “Dr. Ion Cantacuzino” Clinical Hospital, 011437 Bucharest, Romania; 9 Department of Internal Medicine, ‘Sf. Maria’ Clinical Hospital, 011172 Bucharest, Romania

**Keywords:** nailfold capillaroscopy, interstitial lung disease, autoimmune diseases, Raynaud phenomenon

## Abstract

Interstitial lung disease (ILD) is a severe complication of certain connective tissue diseases (CTDs) such as systemic sclerosis (SSc), mixed connective tissue disease (MCTD), idiopathic inflammatory myopathies (IIM), rheumatoid arthritis (RA) and systemic lupus erythematosus (SLE), and it is associated with nailfold videocapillaroscopy (NVC) changes and increased morbidity and mortality rates. Early diagnosis is crucial in order to prevent the progression of ILD, prevent respiratory failure and enhance the patient’s overall quality of life. The most common paraclinical investigations are high-resolution computed tomography (HRCT) and functional respiratory tests such as forced vital capacity (FVC) and the diffusing capacity of the lungs for carbon monoxide (DLCO). The most frequent CTD associated with both ILD and NVC changes is systemic sclerosis. The “late” scleroderma pattern was the most common abnormality identified in NVC results in SSc patients. Other autoimmune diseases were also correlated with ILD and NVC changes, especially when the Raynaud phenomenon was present. Low capillary density was associated with the presence and severity of ILD and a reduction in FVC and DLCO. NVC can also differentiate the capillaroscopic changes in some particular types of ILD, such as the usual interstitial pneumonia (UIP) pattern from the non-specific interstitial pneumonia (NSIP) pattern. Nevertheless, further extensive research is necessary in order to establish the diagnostic value of NVC in CTD-ILD in clinical practice.

## 1. Introduction

**Figure 1:**
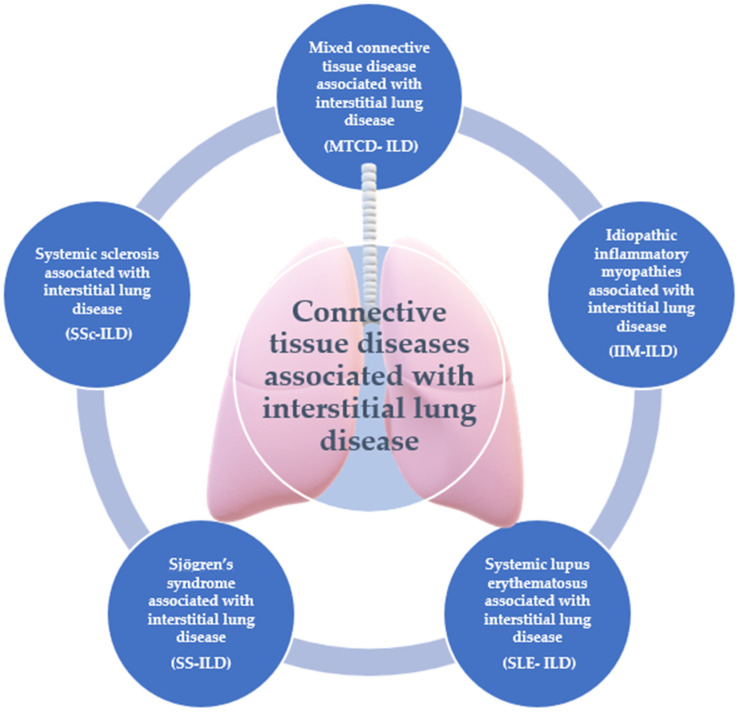
Connective tissue diseases associated with interstitial lung diseases.

The role of nailfold videocapillaroscopy (NVC) in the management of interstitial lung disease (ILD) has not been as well studied compared to pulmonary hypertension, but since ILD can be associated with autoimmune diseases, such as systemic sclerosis (SSc) or other connective tissue diseases (CTDs), such as rheumatoid arthritis (RA), systemic lupus erythematosus (SLE), Sjögren’s syndrome (SS), idiopathic inflammatory myopathies (polymyositis, dermatomyositis), mixed connective tissue disease (MCTD) (Figure 1) and undifferentiated connective tissue disease, the importance of this tool in evaluating patients with ILD deserves further research [1,2,3].

ILD can present itself in various forms; therefore, its correct classification is mandatory because ILD associated with autoimmune diseases requires a completely different form of management. From all CTDs, SSc, RA and myositis are associated with the highest risk of ILD [1,2].

Rheumatologists are the main physicians who use nailfold videocapillaroscopy, which is a non-invasive diagnostic technique, with the role of identifying anomalies in the capillaries of the proximal periungual region of skin on the fingers and toes. This diagnostic tool is particularly useful in systemic sclerosis, and it has been demonstrated that NVC can facilitate the classification of SSc-ILD. Evidence supporting its usefulness has been observed in idiopathic pulmonary fibrosis (IPF) and interstitial pneumonia with autoimmune features (IPAF), although it remains inconclusive [4].

The most common nailfold pattern in SSc-ILD is the “late” scleroderma pattern [4], which is followed by “active” and “early” scleroderma patterns and normal nailfold patterns, in this order. Aberrant nailfold patterns, especially “late” and “active” ones, are correlated to the presence of SSc-ILD [4].

Interstitial lung disease is a common pulmonary manifestation. There are many subgroups within ILD that can be present: idiopathic pulmonary fibrosis (IPF), connective tissue disease–ILD, hypersensitivity pneumonitis, ILD associated with sarcoidosis and other ILDs [5]. Another feature of ILD is interstitial pneumonia with autoimmune characteristics (IPAF), which represents an entity characterized by the presence of ILD and certain autoimmune traits, but it does not meet the exact criteria for an autoimmune disease. This idea was first developed by the European Respiratory Society/American Thoracic Society Task Force, and it is thought to represent the preclinical stages of an autoimmune illness [6].

ILD is more frequent in patients with SSc, but it can also be a presenting feature of other autoimmune illnesses, including inflammatory myositis, rheumatoid arthritis, primary Sjögren’s syndrome, and systemic lupus erythematosus. Microcirculation abnormalities can be detected through nailfold capillaroscopy. Patients with SSc are more likely to exhibit aberrant capillaries and other unusual abnormalities [5,7].

## 2. Normal Capillary Pattern

NVC is a non-invasive technique that is used in the assessment of capillary abnormalities in conditions that fall under the umbrella of collagen diseases, including systemic sclerosis, polymyositis (PM), dermatomyositis (DM), MCTD and SLE. NVC is used to evaluate the changes in the microcirculation, especially in patients with SSc and Raynaud phenomenon, but it is also used as a predictor of complications such as pulmonary hypertension (PH) and interstitial lung disease (ILD). NVC can differentiate primary Raynaud phenomenon (RP) from secondary RP. The clinician should always evaluate the morphology, dimension, density (number of capillaries per linear mm), the presence or absence of hemorrhages and neoangiogenesis. A few tortuosities and crossed capillaries can sometimes be found in healthy individuals [8,9].

Capillary characteristics can be evaluated by measuring them using a unit of measurement, such as linear millimeters. Capillary density (number), morphology (shape), dimension, tortuosity, crossing, the presence or absence of hemorrhages and neoangiogenesis can be evaluated by NVC. There are two NVC patterns. The “scleroderma pattern” is characterized by distinctive capillaroscopic anomalies: the presence of giant capillaries (also known as capillaries with an apical diameter of ≥50 μm) or the combination of aberrant morphologies with a significantly reduced capillary number. Scleroderma capillaroscopic patterns are divided into “early”, “active” and “late”. There is also the “non-scleroderma pattern”, divided into “normal” and “non-specific abnormalities”, which can be present in healthy people or in people with CTDs other than SSc [10]. The “normal pattern” is characterized by capillaries that are hairpin-shaped, equally sized and parallel to one another, and have a perpendicular position. The number of capillary loops per millimeter is expressed by the mean capillary density, which is normally between 7 and 16 capillaries per millimeter [11].

## 3. NVC in Systemic Sclerosis Associated Interstitial Lung Disease (SSc-ILD)

Certain capillaroscopic anomalies, such as giant capillaries, hemorrhages, low capillary density and neoangiogenesis can be found in SSc, PM, DM and MCTD, and even rheumatoid arthritis (RA) and psoriatic arthritis (PsA) [9,12].

Many studies have demonstrated the connection between NVC changes and ILD in patients with SSc (Table 1). A “late” pattern in NVC results was found to be associated with a higher incidence of digital ulcers as well as organ involvement, which was demonstrated by Caramaschi et al. Caetano et al. found that capillary loss and avascular areas were correlated with the presence of ILD and also with a worse respiratory function reflected by a lower FVC and DLCO. Bredemeier et al. discovered a correlation between the avascular area scores from NVC and the activity of ILD identified on high-resolution computed tomography (HRCT), especially in patients with less than five years of disease duration [13]. The identification of micro-hemorrhages, micro-thromboses, giant capillaries and a lower capillary density was correlated with disease activity in systemic sclerosis patients [14].

Other studies also found a significant correlation between the avascular area prevalence in NVC results and the presence of ILD. Marino Claverie et al. showed that in patients with ILD, the “late” sclerodermic pattern is correlated with abnormal respiratory functional tests in patients with SSc. Another study showed that the presence of the “active” pattern is associated with reduced FVC < 70% [8]. SSc patients with a more severe ILD have a lower giant capillaries score [15].

Guillen-del-Castillo et al. showed that there is a relation between capillary loss, avascular regions and neoangiogenesis and the presence of ILD in HRCT results, but also with worse values of respiratory functional tests [16].

Corrado et al. found that microangiopathy changes in NVC results are more severe in patients with SSc-ILD than in patients with idiopathic interstitial lung disease; therefore, microvascular injuries are an important predictor factor in lung fibrogenesis [8].

## 4. NVC in Mixed Connective Tissue Disease Associated with Interstitial Lung Disease (MCTD-ILD)

Mixed connective tissue disease is an autoimmune disorder characterized by the mandatory presence of anti-U1-RNP antibodies and varied clinical manifestations from at least two other collagenases (SLE, SSc, RA), including puffy hands (hand edema), sclerodactyly and RP. MCTD is often associated with severe ILD. ILD in MCTD usually shows an NSIP pattern, followed by UIP, and it occurs in about 50% of patients [17,18]. Studies have shown that the presence of puffy hands, sclerodactyly and giant capillaries identified in NVC results is associated with ILD [8,17].

One study showed that the presence of giant capillaries found using NVC might be an indicator of early ILD, and since almost half of the patients with MCTD develop ILD, NVC should be considered even in patients without respiratory symptoms [8,17].

Another study found that the presence of avascular areas and reduced capillary density in NVC results is associated with ILD (Table 1), particularly in patients with a disease duration of 8.5 years. Moreover, immunosuppressants may improve respiratory manifestations as well as those abnormalities identified with NVC [8,17].

## 5. NVC in Idiopathic Inflammatory Myopathies Associated with Interstitial Lung Disease (IIM-ILD)

ILD is present in 65% of PM/DM patients and is associated with the positivity of MDA5 (melanoma differentiation-associated protein 5) antibodies, especially in severe types of ILD. The most prevalent pattern in HRCT results is NSIP, followed by organizing pneumonia (OP) [19,20].

Antisynthetase antibodies syndrome (ASAS) is an idiopathic inflammatory myopathy characterized by the presence of severe ILD, myositis, arthritis, fever, mechanic’s hands, Raynaud’s phenomenon and the presence of antiaminoacyl tRNA synthetase antibodies (anti-ARS). This syndrome has been associated with a scleroderma pattern in NVC results [21,22].

Studies have shown that NVC might be useful in the diagnosis of IIM, regardless of the presence or absence of Raynaud’s phenomenon, suggesting there can be NVC changes in IIM (Table 1) even in the absence of RP [21].

The presence of bushy capillaries and giant capillaries has been observed in amyopathic forms of IIM. Normal creatine phosphokinase (CK) levels have been associated with ILD. In clinical practice, NVC is not usually used for these patients [21].

Studies reported ramified capillaries as the most common NVC change in ASAS patients, and the SSc pattern was seen in 35.3% of patients. ILD was also associated with ramified capillaries but not with the SSc pattern. One modest research study observed the association of Jo-1 antibodies in IIM patients with a decrease in capillary density [23,24].

In ASAS patients, avascular areas were linked to myositis, but there was no correlation between the presence of arthritis and NVC changes. Also, the main NVC changes have been reported in DM patients, rather than in those with PM. Moreover, the NVC characteristics found in DM at the onset of the disease were giant and ramified capillaries, and, during the course of the disease, the presence of bushy capillaries was reported [23].

One research study that included patients with PM and DM, conducted by Selva-O’ Callaghan et al., found a significant correlation between the quantification of NVC changes, disease activity and severity and the presence of ILD and RP. No association between disease duration and the quantification of NVC changes was found [25].

## 6. NVC in Systemic Lupus Erythematosus Associated with Interstitial Lung Disease (SLE-ILD)

SLE is a chronic systemic autoimmune disease that can involve almost any organ and is characterized by the presence of specific serum antibodies and the deposition of immune complexes. Lung disease can be present in SLE, and the most prevalent manifestation is represented by pleurisy, which affects 60% of patients. ILD is present in 15% of patients, and the most common pattern identified in HRCT results is NSIP. SLE-ILD has a better prognosis than idiopathic ILD [18].

Some NVC changes can be found in SLE patients, but they are less frequent compared to SSc and are correlated with disease activity. Studies have shown that patients with NVC abnormalities such as hemorrhages had an active disease (reflected by the SLEDAI score), and those with inactive disease had normal capillaroscopic findings. The main NVC changes identified in SLE patients were non-specific patterns represented by erythrocyte aggregation and capillary dilatation (Table 1). The normal pattern was less frequent, followed by the scleroderma pattern, which was associated with RP [26].

One study showed that the non-specific pattern was identified in 56.6% of SLE patients, while the scleroderma pattern and the normal pattern were found in 9.2% and 34.2%, respectively [27].

## 7. NVC in Sjögren’s Syndrome (SS) Associated with ILD

Sjögren’s syndrome (SS) is a chronic autoimmune disease that particularly involves the exocrine glands and is characterized by Sicca syndrome. In addition to affecting the lacrimal and salivary glands, it can present with variate systemic manifestations, such as RP and lung disease. A few studies reported NVC changes identified in primary SS (Table 1), such as lower capillary density and dilated capillaries, especially when associated with RP (10–30% of cases). Lower capillary density was also correlated with ILD in SS [18,28,29]. ILD appears in 9–20% of SS patients, and the most prevalent pattern on HRCT is represented by NSIP (45%), while the second and third places are occupied by UIP and lymphocytic interstitial pneumonia (16% and 15%) [18].

The scleroderma pattern can also be present in an overlap syndrome with systemic sclerosis or mixed connective tissue disease [29].

## 8. Discussion

ILD is a pulmonary complication of CTDs, which are more common in women, and it is associated with increased morbidity and mortality rates; therefore, early diagnosis is mandatory in order to prevent respiratory failure. In addition to high-resolution computed tomography (HRCT), functional respiratory tests such as FVC and DLCO (the diffusing capacity of the lungs for carbon monoxide) are used to evaluate ILD. FVC has a low value in restrictive disorders such as pulmonary fibrosis, whereas a low DLCO value represents a membrane diffusion incapacity. NVC is often used in SSc patients, but it was observed that NVC might have an important role in other CTDs, and a low capillary density was associated with the presence and severity of ILD and a reduction in FVC and DLCO [30,31].

**Figure 2:**
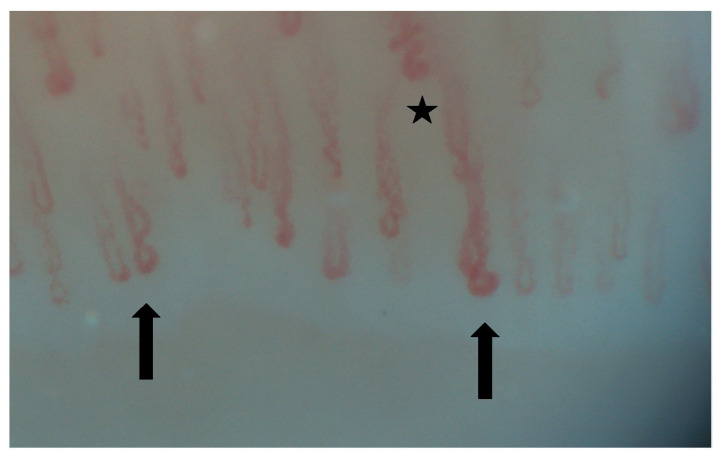
Tortuous (*) and crossed capillaries (↑).

**Figure 3:**
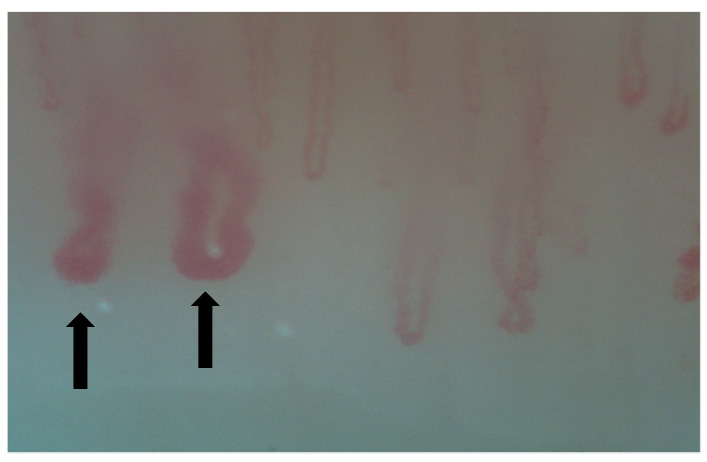
Giant capillaries.

**Figure 4:**
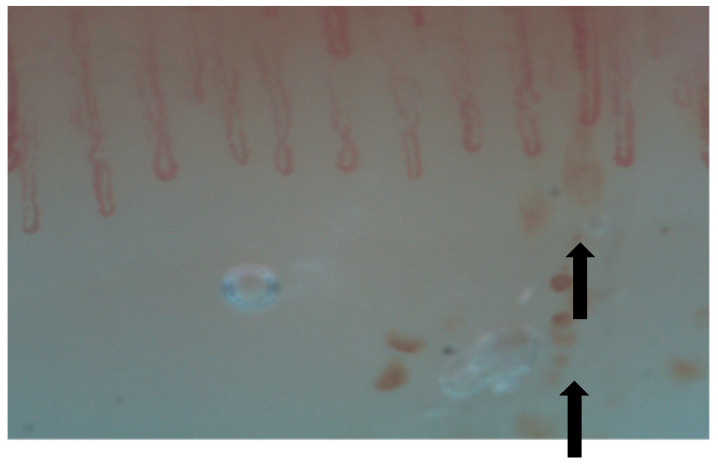
Micro-hemorrhages.

**Figure 5:**
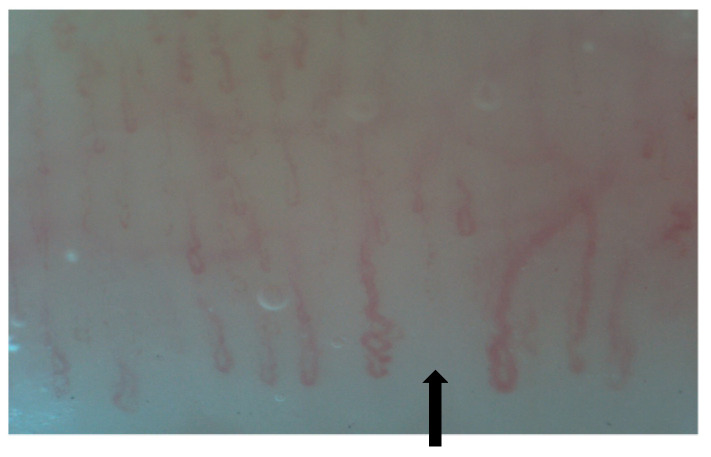
Avascular area.

**Figure 6:**
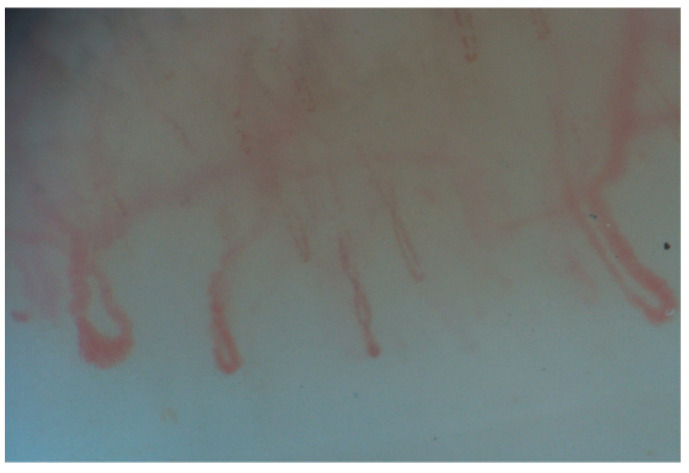
“Early” scleroderma pattern.

**Figure 7:**
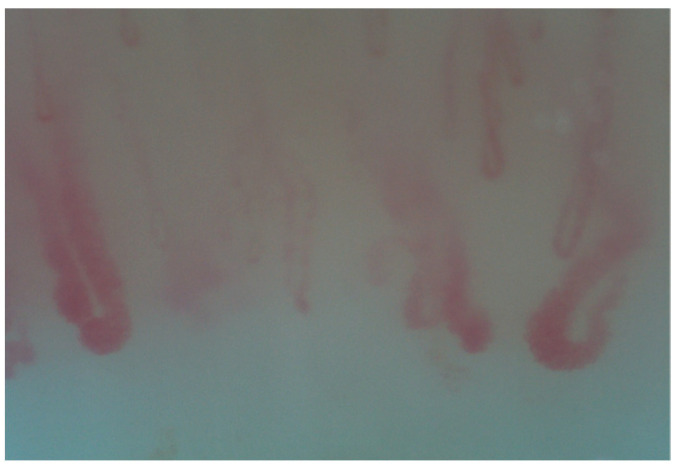
“Active” scleroderma pattern.

Nailfold videocapillaroscopy (NVC) is a simple, non-invasive technique that can be used for the screening of systemic sclerosis’s complications, such as organ involvement, mainly represented by interstitial lung disease (ILD). This tool can identify early microvascular changes in patients with secondary Raynaud phenomenon (Figure 2, Figure 3, Figure 4, Figure 5, Figure 6 and Figure 7). It may also be helpful in other CTDs, such as MCTD, RA, IIM, SLE, etc. [13,32,33,34]. Hysa et al. showed that patients with CTDs such as SSc, MCTD and IIM had non-specific changes and giant capillaries in NVC results more commonly that patients with other CTDs [33].

Caetano et al. showed that there is a significant correlation between capillary loss and avascular areas in NVC results and the presence and severity of ILD. The majority of individuals included in this study had an active pattern in their NVC results, contrary to others, who showed a higher prevalence of the late pattern [13].

Umashankar et al. showed that the highest prevalence of nailfold videocapillaroscopic abnormalities is found in ILD associated with autoimmune diseases (80.4%); therefore, it seems that NVC is more useful in CTDs-ILD (connective tissue diseases associated with interstitial lung disease), rather than in idiopathic pulmonary fibrosis (IPF). Umashankar et al. also showed that SSc-ILD is correlated with a high prevalence of scleroderma patterns (early, active or late) in NVC results. As a result, screening for the onset of ILD is recommended in SSc patients with suggestive patterns in NVC results. Furthermore, this procedure could be also performed in the future for other CTDs, such as myositis or mixed connective tissue disease (MCTD) [1].

Rudra et al. showed that there is a significant correlation between NVC abnormalities and the severity of ILD and respiratory symptoms in SSc patients. All SSc patients can benefit from NVC, in addition to clinical evaluation (lung auscultation), functional respiratory tests and chest radiography, in order to identify early ILD before the onset of respiratory symptoms [35].

Standardizing the criteria used for the evaluation of nailfold capillary anomalies is crucial for future research. This will enable a deeper comprehension of NVC’s usefulness in ILD [4].

Some authors consider that NVC might be a useful technique in patients with MCTD with a short disease duration in order to evaluate the presence of giant capillaries, which were associated with severe ILD. The evolution of ILD in MCTD has a slow course compared to the one associated with SSc or IIM [8,17].

Avascular areas in NVC results are also associated with the presence of ILD. The late pattern found in NVC results was associated with abnormal respiratory functional tests in patients with SSc-ILD. The active pattern in NVC results is associated with reduced forced vital capacity (FVC) <70% [8]. In SLE patients, the most common NVC finding was the non-specific pattern. A percentage between 18 and 40% of SLE patients have RP, which was correlated with a scleroderma pattern and a higher risk of organ involvement, including lung disease (interstitial pneumonia and pulmonary hypertension). Treatment initiation was correlated with an improvement in capillaroscopic changes [26].

There are few studies that reported the NVC abnormalities associated with ILD in diseases other than SSc. Regarding SS, the NVC findings were reported mainly in primary SS, and more research is necessary in order to establish that these patients have particular NVC changes [29].

Interstitial pneumonia with autoimmune features (IPAF) is a condition that associates ILD and different characteristics of autoimmunity but does not meet the criteria for a specific connective tissue disease. CTDs are one of the main causes of ILD, accounting for up to 30% of newly diagnosed cases; therefore, it is advised to rule out the possibility of a CTD [36].

Specialty literature demonstrated that 13.5–18.8% of cases with IPAF will progress to a specific CTD. One study, that compared the different patterns identified in HRCT results from patients with CTD-ILD, IPAF and IIP, demonstrated that the most frequent pattern in patients with CTD-ILD and IPAF is the non-specific interstitial pneumonia pattern, compared to IIP, which is associated with usual interstitial pneumonia pattern. Moreover, CTD-ILD and IPAF were correlated with the presence of giant capillaries and hemorrhages in NVC results. The scleroderma pattern was also identified in NVC results in 27.8% of IPAF patients, although it was more frequent in CTD-ILD cases [5].

Another study showed that the usual interstitial pneumonia pattern in HRCT results was correlated with a higher frequency of tortuous capillaries compared to non-specific interstitial pneumonia in both CTD-ILD and non-CTD ILD cases. NVC might be a useful technique to diagnose a potential CTD in ILD patients [37].

Another role of NVC is to differentiate the alterations from some particular types of ILD, such as the usual interstitial pneumonia (UIP) pattern from the non-specific interstitial pneumonia (NSIP) pattern [38].

The identification of patients at high risk of developing or the progression of ILD is mandatory since the course of this disease can be fatal due to high mortality and morbidity rates. Those patients at high risk of disease progression can be treated before their lung function deteriorates, meaning that regular screening should be performed in order to identify pulmonary changes suggestive of ILD or ILD progression [39].

Although there is no biomarker for predicting the evolution of ILD in SSc patients, some risk factors of ILD progression were identified for better management of these patients. Diffuse cutaneous sclerosis, the presence of anti-topoisomerase I antibodies, anti-CXCR3 and anti-CXCR4 antibodies, older age, Afro-American ethnicity, low FVC or DLCO and the extent of ILD in HRCT results were all associated with the progression of SSc-ILD [40,41].

One recent study evaluated the capillaroscopic changes in CTDs-ILD and non-CTD ILD patients and the impact of these changes on the pathophysiological mechanisms, diagnosis, treatment and prognosis of these diseases. This study showed that patients with non-CTD ILD, especially those with UIP found through chest radiography, presented with more tortuous capillaries through NVC [37]. This result might represent the fact that there are microvascular abnormalities in patients with pulmonary fibrosis, underlying the fact that angiogenesis and microvascular dysfunction have a role in the pathophysiology of non-CTD ILD [37,42].

Moreover, it is known that fibroblasts and extracellular matrix accumulation is a feature of disorders associated with pulmonary fibrosis. On the other hand, patients with CTDs showed a non-specific capillaroscopic pattern [37,43].

The mechanisms underlying the correlation between microvascular damage identified by NVC and ILD are still unclear. The initial process is the microvascular injury. It is thought that both endothelial and epithelial injuries lead to inflammation and consecutive fibrosis. The proinflammatory mediators implicated in the physiopathology of ILD and SSc-ILD are represented mainly by transforming growth factor beta (TGFβ), platelet-derived growth factor, endothelin-1 (ET-1) and vascular endothelial growth factor, which eventually lead to microvascular dysfunction due to increased vascular permeability and the activation of fibroblasts. The angiopoietin system is also responsible for the vascular barrier, especially ANGPT1 (angiopoietin 1), which maintains the endothelium’s stability [44,45,46,47].

Regarding the prognosis, Lee et al. used the GAP (gender, age, physiology) score to predict mortality in patients with idiopathic pulmonary fibrosis and showed that the group with a poor prognosis had more capillaroscopic changes defined by an SSc pattern. On the contrary, the good prognosis group did not show any of these capillaroscopic abnormalities [48]. These results might suggest that NVC might have a role in predicting the course of the disease, since the degree of pulmonary fibrosis is directly correlated with the number of NVC abnormalities. Therefore, interstitial lung disease could be detected in the early stages; studies suggest that all patients with ILD should perform NVC, corroborated by immunological profiles as well as imaging and respiratory functional tests in order to make an early diagnosis of CTD and initiate immunosuppressive and/or antifibrotic treatment [37,48].

## 9. Future Directions

NVC might be a useful tool to predict lung complications in patients with autoimmune diseases, including SSc, MCTD, SLE, SS, IIM, rheumatoid arthritis and psoriatic arthritis [48,49]. Evidence showed that NVC changes in SSc patients were correlated with a high risk of lung complications such as ILD. NVC might be included in prediction systems for early recognition of lung complications in SSc patients, together with other parameters. Also, lung ultrasound is considered a safe, non-invasive tool that might be useful in the future for detecting ILD [50,51,52]. Since there are not many available studies worldwide regarding NVC and its potential role in the early detection of microvascular changes correlated to CTDs-ILD, more research, including a larger amount of patients, would be helpful in order to better understand the role of NVC in evaluating and managing CTD patients. The non-invasive nature of NVC makes this procedure easy to perform and essential in the management of patients with CTD-ILD [2].

By addressing the current state of knowledge, the paper identifies gaps in the research and suggests directions for future studies. This is essential for advancing the field and improving diagnostic accuracy. Overall, this comprehensive review plays a crucial role in bridging the gap between research and clinical application.

## 10. Conclusions

Since numerous studies have shown that a significant correlation between NVC changes, respiratory functional tests (FVC and DLCO) and the presence of ILD in HRCT results exists, it is plausible to believe that microangiopathy is a major factor in the initiation and progression of ILD, particularly SSc-ILD.

Further extensive research is necessary in order to establish the diagnostic value of NVC in CTD-ILD in standard medical practice, and in the meantime, high-resolution CT currently remains the gold-standard tool for the diagnosis of SSc-ILD.

These correlations between microvascular changes identified in NVC results and the presence of ILD suggest that vascularization alterations might be the underlying physiopathological process of ILD.

## Figures and Tables

**Table 1:** NVC abnormalities reported in CTDs associated with ILD.

	SSc-ILD	MCTD-ILD	IIM-ILD	SLE-ILD	SS-ILD
NVC abnormalities	₋ late pattern (capillary loss, avascular regions and neoangiogenesis) ₋ micro-hemorrhages, micro-thromboses, giant capillaries and a lower capillary density correlated with active disease	₋ giant capillaries, capillary loss, avascular area	₋ giant and ramified capillaries at the onset of the disease ₋ bushy capillaries during the course of the disease	₋ non-specific pattern, most frequently (erythrocyte aggregation, capillary dilatation) ₋ normal and scleroderma pattern, less frequently	₋ lower capillary density ₋ scleroderma pattern in overlap syndromes

## Data Availability

All available information was presented in this paper.
